# Hydrotherapy to Hospice: The Hidden Perils of Necrotizing Fasciitis Mimicry in Elderly Patients

**DOI:** 10.7759/cureus.74221

**Published:** 2024-11-22

**Authors:** Moustafa Elramlawy, Ahmed H Abdellatif, Hany Abdelmasih, Karim Hussien

**Affiliations:** 1 Emergency Department, Stoke Mandeville Hospital, Aylesbury, GBR; 2 Medicine for Elderly People, Stoke Mandeville Hospital, Aylesbury, GBR; 3 Acute Medicine, Stoke Mandeville Hospital, Aylesbury, GBR; 4 Trauma and Orthopaedics, Buckinghamshire Healthcare NHS Trust, Stoke Mandeville Hospital, Aylesbury, GBR

**Keywords:** acute kidney injury(aki)), elderly falls, gas gangrene, necrotizing fasciitis, opioid overdose, severe sepsis

## Abstract

This case report presents a 77-year-old female with dementia and hypertension who experienced reduced consciousness during hydrotherapy. She was found to have hypotension, a low Glasgow Coma Scale score, and right thigh pain. Blood work showed acute kidney injury and elevated inflammatory markers, while imaging revealed surgical emphysema in the right thigh and pelvis without trauma.

Necrotizing fasciitis was ruled out, but anaerobic cultures identified Bacteroides fragilis, suggesting non-clostridial gas gangrene from a suspected colonic perforation. Due to clinical instability and comorbidities, surgical intervention was not feasible. Despite antibiotics and supportive care, her condition deteriorated, and she transitioned to palliative care. This case highlights the diagnostic challenges in complex elderly presentations.

## Introduction

Non-clostridial gas gangrene is a rare but serious infection that presents unique diagnostic and management challenges. Unlike clostridial gas gangrene, which is often associated with traumatic injury and the presence of Clostridium species, non-clostridial gas gangrene typically involves other anaerobic bacteria, such as Bacteroides fragilis, and can arise without trauma, frequently due to underlying gastrointestinal pathology. Bowel perforation can introduce these bacteria into surrounding tissues, leading to rapid gas formation, severe soft tissue infection, and potential systemic toxicity. Early diagnosis can be challenging, however, as non-clostridial gas gangrene may present without overt skin changes or other classic signs of infection. This subtle presentation, combined with the high morbidity and mortality associated with delayed treatment, underscores the importance of maintaining a high index of suspicion in elderly or immunocompromised patients presenting with abdominal pain and gas in soft tissues on imaging. Early multidisciplinary involvement, including surgery, infectious diseases, and radiology, is essential to improving outcomes in these complex cases [1-2].

## Case presentation

A 77-year-old female was brought by ambulance to the ED after suddenly falling asleep in the pool during her regular hydrotherapy session. She remained drowsy, with a reduced level of consciousness, prompting the ambulance to transport her urgently to the ED. At the time of the incident, she was accompanied by her trainer, effectively ruling out the possibility of a fall or near-drowning during the session. According to the ambulance crew’s assessment, the patient was initially hypotensive and required 4 liters of oxygen to maintain normal oxygen saturation. Her past medical history includes dementia, essential hypertension, a stable aneurysm in the right internal carotid artery, and osteoarthritis in the right knee, for which she attends hydrotherapy. Her medications include bendroflumethiazide, codeine, donepezil, and lisinopril.

Upon arrival, during the primary survey, a nasopharyngeal airway was inserted in her left nostril to optimize oxygenation. Chest auscultation was normal, with a heart rate of 70 beats per minute and blood pressure of 90/50 mmHg. Capillary refill was 4 seconds. Her Glasgow Coma Scale (GCS) score was 9/15 (Eye: 2, Voice: 2, Motor: 5), and she had pinpoint pupils. Her temperature was 35.5°C, and her blood sugar was normal. On abdominal examination, she exhibited tenderness in the right lower quadrant, extending to the right inguinal region. The patient also localized pain to the right thigh, raising clinical suspicion of a neck of femur fracture. The patient was treated with multiple intravenous naloxone injections, each resulting in temporary improvement in her level of consciousness. However, she continued to have a poor level of consciousness, so a naloxone infusion was initiated. She also received IV amoxicillin to cover for possible sepsis.

One of the main suspected causes of the patient's presentation was a possible opioid overdose. However, upon further questioning, the patient’s daughter confirmed that the medications are stored in a locked cabinet, and she is the sole administrator, greatly reducing the likelihood of an overdose. The daughter also noted that the patient had been limping for the past two days due to right leg pain and had experienced a fall on the day of presentation, though there was no significant trauma or change in mobility following the incident.

Investigation

Given the patient's history and presentation, blood samples were collected for a full blood count, liver profile, kidney profile, C-reactive protein, creatine kinase, and lactate. Results showed a significant elevation in infection markers (Table 1). Both creatinine and urea levels were elevated, with no prior history of renal impairment, indicating an acute kidney injury. A venous blood gas was done, and it was unremarkable with a lactate level of 1.6 mmol/L and normal electrolytes.

**Table 1:** Blood results indicating elevated levels of CRP, CK, WCCs, and neutrophils. Additionally, there is an increase in urea and creatinine levels, suggesting acute kidney injury. CRP: C-Reactive Protein; CK: Creatine Kinase; WCCs: White Cell Counts; CKD: Chronic Kidney Disease.

Test	Result	Reference
CRP	>320	0-5 mg/L
Creatine Kinase	809	29-168 U/L
WCC	23.0	3.7-11 10^9^/L
Abs Neutrophils	21.1	1.7-7.5 10^9^/L
Platelets	461	150-450 10^9^/L
Sodium	136	136-145 mmol/L
Potassium	4.4	3.5-5.1 mmol/L
Urea	21.6	2.5-6.7 mmol/L
Serum Creatinine	188	50-98 µmol/L
Alanine Transferase	18	10-35 U/L
Alkaline Phosphatase	102	40-150 U/L
Total Bilirubin	7	0-21 µmol/L
Lactate	1.8	<1 mmol/L

**Figure 1:**
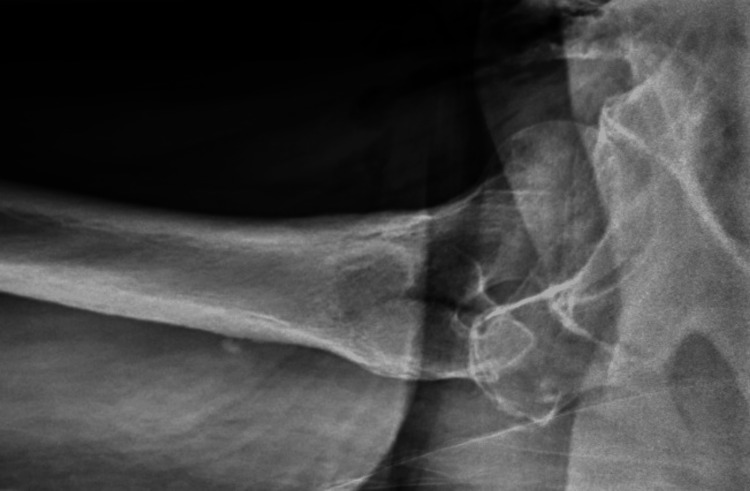
X-ray of the right hip showing no fracture, with surgical emphysema.

**Figure 2:**
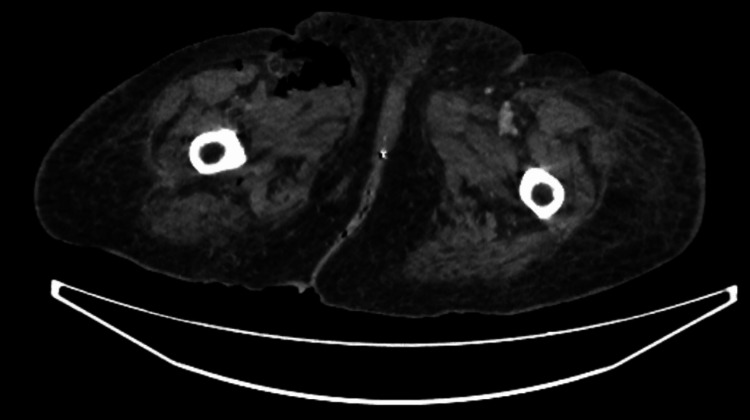
CT scan of both lower limbs showing gas in the right thigh.

**Figure 3:**
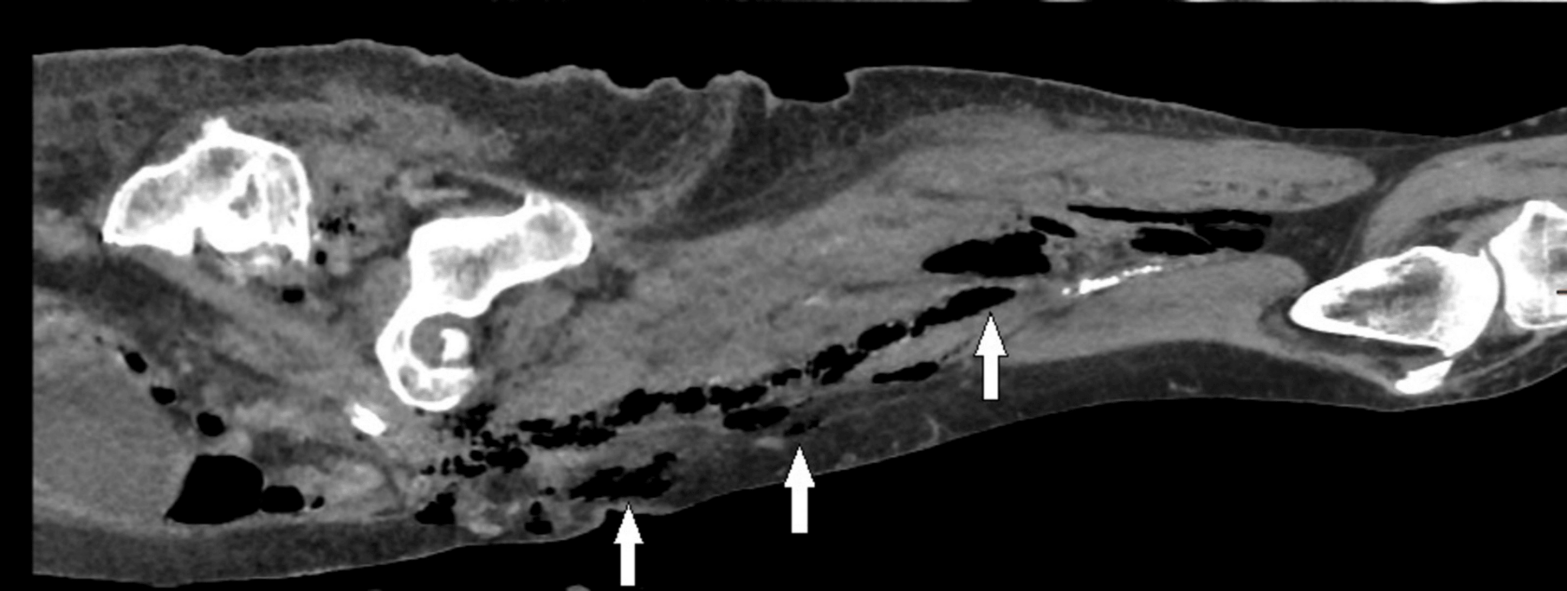
Sagittal plane CT scan of the right thigh showing tracking of surgical emphysema.

Given the history of a fall and the unclear cause of her low GCS, a CT trauma series was requested, along with an X-ray of the right hip to rule out injuries. The right hip X-ray showed no fracture; however, surgical emphysema was noted (Figure 1). The CT of the head was unremarkable, showing a stable, unchanged aneurysm and no fractures in the cervical spine. A CT scan of the chest, abdomen, and pelvis revealed surgical emphysema in the right thigh, gluteal region, and pelvis, without evidence of associated traumatic injury (Figures 2-3).

The right piriformis muscle appeared slightly enlarged and contained gas. The sigmoid colon showed extensive diverticular disease. Significant gas was noted on the right side of the abdomen, though no definitive source of free gas was identified, with the sigmoid colon as a possible origin. While no collections were seen that would be suitable for drainage, extensive gas was observed tracking through the deep right pelvis, extending anteriorly and laterally. A small perforation of the adjacent sigmoid colon could not be entirely ruled out, as it was positioned near the tracking gas locules.

Outcome 

The patient was initially treated in the Emergency Department for an infection of unknown origin with concurrent acute kidney injury. Potential sources of infection included an abdominal source or necrotizing fasciitis. She was started on Tazocin and clindamycin. The plastics team was consulted as the patient had a Laboratory Risk Indicator for Necrotizing Fasciitis (LRINEC) score of 9. A plastic surgeon performed a diagnostic sweep test of the right groin, which showed an intact fascia lata with no pus present. A sample was collected from the thigh, and results indicated no organisms were seen (Table 2).

**Table 2:** Results of the fluid collected during the Sweep test of the right thigh.

Item	Finding
Macroscopic appearance	Slightly blood-stained fluid
Gram film: Pus cells	Scanty
Organisms	No organisms seen

Based on these findings, the plastics team deemed the sweep test negative, making necrotizing fasciitis a less likely diagnosis. Concerned about a possible abdominal source, they requested an evaluation by the general surgery team. The patient was referred to general medicine for admission with input from general surgery.

The general surgery team reviewed the patient and expressed concern about a possible colon perforation, which could not be definitively ruled out by the CT scan. However, the patient was too unstable to undergo a laparotomy due to her high NEWS score and advanced age. Their initial plan was to switch antibiotics to co-amoxiclav, metronidazole, and gentamicin, aiming to stabilize her condition as much as possible in preparation for surgery if needed. If no improvement was observed, a repeat CT scan would be considered.

**Figure 4:**
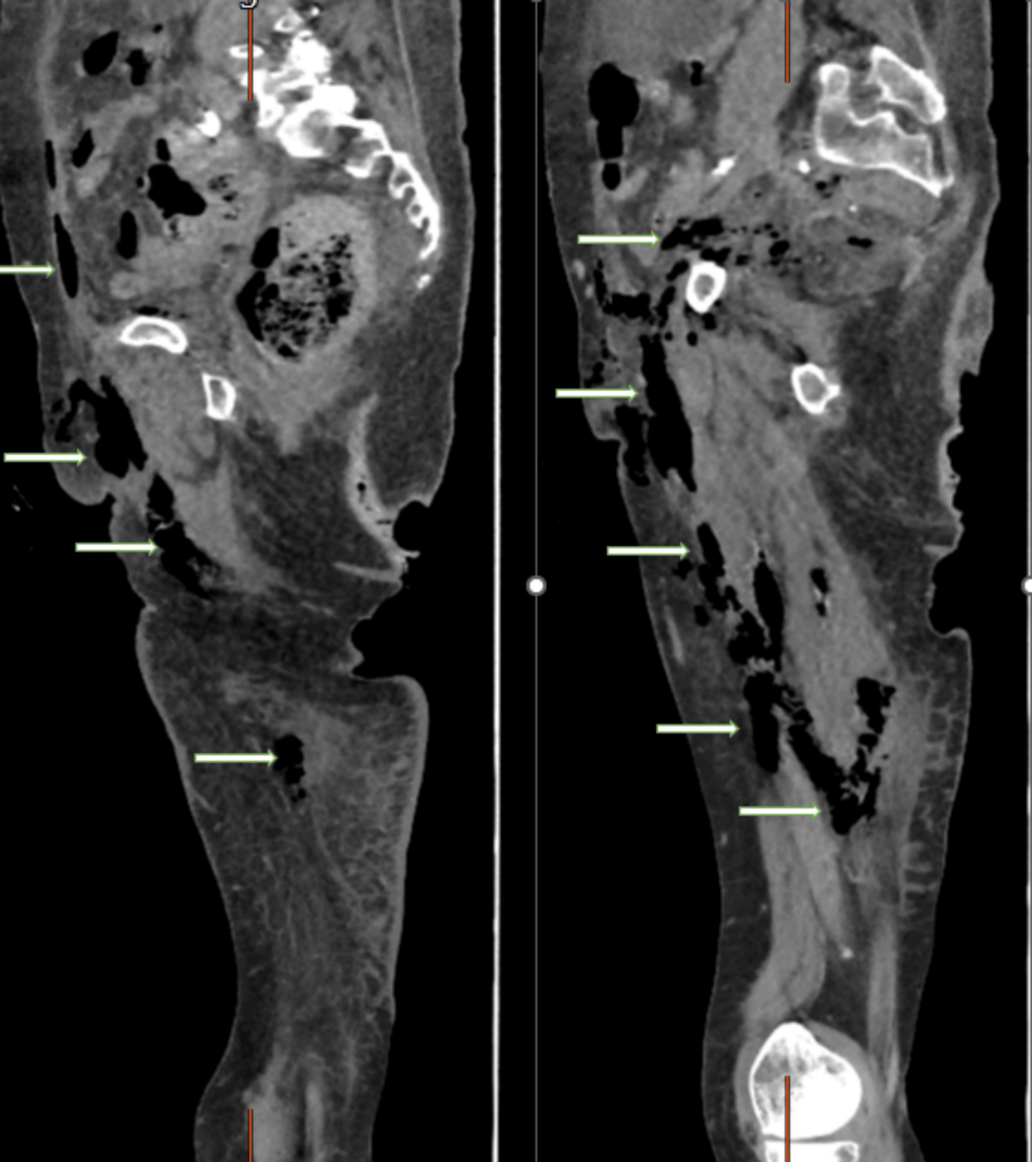
Sagittal plane of a CT scan showing surgical emphysema, indicated by arrows.

As the patient’s condition did not improve, another CT scan was performed, revealing the following findings: extensive subcutaneous emphysema tracking through the anterior abdominal wall, right inguinal region, perineum, right pelvic sidewall, and extending along the right adductor and hamstring compartments. There was overlying subcutaneous edema, but no focal collection was identified. These findings raised suspicion for necrotizing fasciitis, gas gangrene, or recent penetrating trauma, and correlation with clinical history and examination was advised (Figure 4).

Since the patient had no history of penetrating injury and necrotizing fasciitis had already been ruled out by the plastics team, the general surgery team decided against surgical intervention for the time being, as she remained unfit for surgery. They opted to continue medical management with antibiotics. Four days later, the anaerobic culture of the fluid sample collected from the sweep test returned positive for Bacteroides fragilis (Table 3). Bacteroides fragilis is an anaerobic, Gram-negative bacterium commonly found in the human GI tract, particularly in the colon, which supports the diagnosis of non-clostridial gas gangrene secondary to a small colonic perforation that was not clearly visible on the CT scan.

**Table 3:** Culture results of the fluid obtained from the sweep test.

Item	Finding
Aerobic culture	Staphylococcus epidermidis
Clarithromycin	Sensitive
Flucloxacillin	Sensitive
Fusidic acid	Resistant
Penicillin	Resistant
Gentamicin	Sensitive
Anaerobic culture	Bacteroides fragilis
Co-amoxiclav	Sensitive
Metronidazole	Sensitive

Despite treatment, the patient showed no improvement, with persistently elevated CRP levels above 290 mg/L and low blood pressure. She was not fit for intensive care due to her age and the poor prognosis. Subsequently, she was deemed unfit for further surgical or medical intervention. After discussions with the patient’s family, they agreed to involve the palliative care team. The patient was commenced on anticipatory medications and transferred to a hospice care facility, where, sadly, she passed away.

## Discussion

This case involved gas gangrene, a severe infection that presented without the typical signs of trauma or skin changes, complicating the diagnosis. The infection resulted in the breakdown of muscles in the anterior abdominal wall and the right thigh, which explains the elevated creatine kinase (CK) levels. Acute kidney injury (AKI) occurred due to either the elevated CK levels or the infection itself. The patient was taking codeine for ongoing osteoarthritis in her right knee; however, this medication is excreted by the kidneys. Due to the AKI, codeine was not effectively excreted, leading to its accumulation in the bloodstream and causing pinpoint pupils and a low Glasgow Coma Scale (GCS) score that mimicked an opioid overdose.

In cases of low GCS with suspected intracranial bleeding (ICB), pupils are typically unequal and do not respond to naloxone, as observed in our patient. However, we cannot completely rule out ICB, making a CT scan of the head essential. Additionally, the sweep test is crucial for diagnosing necrotizing fasciitis. Furthermore, the patient was having a temperature of 35.5°C with a lactate level of 1.6 mmol/L in the venous blood gas; clinicians might think that it is not an infection, but the patient was actually experiencing hypothermic sepsis, which is more dangerous and fatal as it could delay the administration of antibiotics.

In the literature review, non-clostridial gas gangrene has been reported in several studies. Kaur M et al. described a case of a non-diabetic patient who developed non-clostridial gas gangrene following a traumatic injury. This case emphasizes the potential for polymicrobial infections to present similarly to classical gas gangrene [3]. Reddy ER et al. reported a patient who developed gas gangrene as a complication following colonic perforation. This case underscores the serious nature of gas gangrene as a potential complication of colonic perforations. The authors emphasize the need for early recognition and treatment to improve patient outcomes [4]. Tasaki M et al. detailed a case of gas gangrene secondary to diverticulitis of the colon, where the perforation extended into the retroperitoneal space. This case illustrates the complications associated with diverticulitis, particularly the risk of gas gangrene when perforation occurs. The authors stress the importance of timely diagnosis and treatment to improve outcomes in similar cases [5].

Sasaki T et al. reported six autopsy cases of non-traumatic gas gangrene in the abdomen. None of the patients had a clinical diagnosis of gas gangrene prior to their deaths. The authors recommend that gas gangrene should be considered in any patient presenting with abdominal infections [6]. Stoyanov GS et al. presented two autopsy cases of primary abdominal gas gangrene. Unlike classical gas gangrene or myonecrosis, this disease develops without a wound or port of entry. Instead, gas-producing bacteria in the gastrointestinal tract colonize an underlying pathological process with foci of necrosis, leading to excessive gas production and hematogenous spread to other organs [7].

Wyman AL et al. discussed a patient who developed endogenous gas gangrene as a complication of colon cancer [8]. A case report by Bessman AN and Wagner W detailed 48 cases of non-clostridial gas gangrene, focusing on the clinical presentation, diagnosis, and management of the condition. Unlike typical clostridial infections, non-clostridial gas gangrene is associated with various organisms, including *E. coli* and Bacteroides. The authors highlight the importance of early recognition and aggressive treatment, which often involves surgical intervention and antibiotic therapy [9].

Learning points

This case highlights the challenges clinicians face in diagnosing non-clostridial gas gangrene, as it can present with few or no obvious clinical signs and may appear atypically without skin changes. Furthermore, it underscores the high mortality and poor prognosis associated with gas gangrene in elderly patients, emphasizing the significance of early recognition and treatment. Additionally, this case emphasizes the importance of ruling out acute kidney injury in patients on opioids who present with clinical symptoms suggestive of an opioid overdose, even with confirmed adherence to prescribed doses. It also illustrates the necessity of prompt and continuous involvement of various specialties, such as general surgery and plastic surgery teams, in cases of suspected gas gangrene or necrotizing fasciitis, particularly when an abdominal perforation is suspected.

The case further highlights the challenges radiologists encounter in diagnosing abdominal gas collections, as gas can obscure visibility and lead to missed perforations on CT scans. Another crucial insight is that wound cultures do not provide 100% sensitivity in ruling out gas gangrene. Finally, it is essential to note that aneurysmal intracranial bleeds are more likely to cause irreversibly unequal, non-reactive pupils, rather than the reversible pinpoint pupils that respond to naloxone. The involvement of family members in clarifying the patient’s baseline status, recent events, and medication adherence proved invaluable to the clinical team, reinforcing the need for effective communication with caregivers in complex cases. Balancing aggressive treatment against a patient’s surgical risk profile requires careful multidisciplinary planning and communication with the family, especially in cases where the infection is advanced and surgical intervention poses high risks.

## Conclusions

This case underscores the value of collaboration among specialties in complex multisystem presentations, where rapid deterioration limits diagnostic and treatment windows. The input from plastic and general surgery teams, combined with serial imaging and culture analysis, was essential in refining the diagnosis. The findings ultimately emphasized that non-clostridial gas gangrene can present insidiously, often with minimal initial symptoms beyond gas in soft tissue and non-specific infection markers.

Clinicians should consider colonic perforation in elderly patients with surgical emphysema and no external injury, particularly in those with underlying diverticular disease or abdominal pathology. Timely, comprehensive evaluation and management are critical for improving outcomes. Additionally, this case highlights the potential for opioid medications to complicate clinical presentations in elderly patients with pre-existing dementia. The initial suspicion of opioid overdose due to her codeine prescription contributed to the challenge of diagnosing the underlying source of infection. This emphasizes the importance of a holistic approach to medication review and differential diagnosis in patients presenting with altered consciousness, especially when opioid toxicity and infection can produce overlapping symptoms.

In conclusion, this case illustrates the necessity of a comprehensive understanding of the physiological and pathological changes associated with gas gangrene. The differences between clostridial and non-clostridial gas gangrene can create significant diagnostic challenges. Clinicians must be aware that seconds count in such cases; early intervention can be life-saving.
